# Induction of photosynthesis under anoxic condition in *Thalassiosira pseudonana* and *Euglena gracilis*: interactions between fermentation and photosynthesis

**DOI:** 10.3389/fpls.2023.1186926

**Published:** 2023-07-25

**Authors:** Gwenaëlle Gain, Nicolas Berne, Tom Feller, Damien Godaux, Ugo Cenci, Pierre Cardol

**Affiliations:** ^1^InBioS – PhytoSYSTEMS, Laboratoire de Génétique et Physiologie des Microalgues, ULiège, Liège, Belgium; ^2^Unité de Glycobiologie Structurale et Fonctionnelle, Université de Lille, CNRS, UMR8576 – UGSF, Lille, France

**Keywords:** *Euglena gracilis*, *Thalassiosira pseudonana*, fermentation, photosynthesis, hydrogenase, cyclic electron flow (CEF)

## Abstract

**Introduction:**

In their natural environment, microalgae can be transiently exposed to hypoxic or anoxic environments. Whereas fermentative pathways and their interactions with photosynthesis are relatively well characterized in the green alga model *Chlamydomonas reinhardtii*, little information is available in other groups of photosynthetic micro-eukaryotes. In *C. reinhardtii* cyclic electron flow (CEF) around photosystem (PS) I, and light-dependent oxygen-sensitive hydrogenase activity both contribute to restoring photosynthetic linear electron flow (LEF) in anoxic conditions.

**Methods:**

Here we analyzed photosynthetic electron transfer after incubation in dark anoxic conditions (up to 24 h) in two secondary microalgae: the marine diatom *Thalassiosira pseudonana* and the excavate *Euglena gracilis*.

**Results:**

Both species showed sustained abilities to prevent over-reduction of photosynthetic electron carriers and to restore LEF. A high and transient CEF around PSI was also observed specifically in anoxic conditions at light onset in both species. In contrast, at variance with *C. reinhardtii*, no sustained hydrogenase activity was detected in anoxic conditions in both species.

**Discussion:**

Altogether our results suggest that another fermentative pathway might contribute, along with CEF around PSI, to restore photosynthetic activity in anoxic conditions in *E. gracilis* and *T. pseudonana*. We discuss the possible implication of the dissimilatory nitrate reduction to ammonium (DNRA) in *T. pseudonana* and the wax ester fermentation in *E. gracilis*.

## 1 Introduction

Some microalgae must cope with long or frequent hypoxic (low oxygen) or anoxic (no oxygen) events in many natural environments such as marine sediments, eutrophic standing shallow waters or ice (Drew, 1997; Banti et al., 2013; Catalanotti et al., 2013). This implies that these organisms are endowed with metabolic capacities such as fermentative pathways and associated electron acceptors which ensure the maintenance of the energy balance (ATP, NAD(P)H) in the dark, and in the light to restore photosynthetic activity. The green unicellular alga *Chlamydomonas reinhardtii* is the microalgal species whose fermentative pathways have been best characterized (Catalanotti et al., 2013). In anoxic conditions, *C. reinhardtii* synthesizes formate, acetate and ethanol as major terminal products (Kreuzberg, 1984; Mus et al., 2007) and some minor products such as hydrogen, lactate, glycerol and carbon dioxide (Atteia et al., 2013; Catalanotti et al., 2013). Three enzymes allow pyruvate conversion into acetyl-CoA: (i) the pyruvate dehydrogenase complex in aerobic conditions and (ii) the pyruvate formate lyase (PFL) and the pyruvate ferredoxin oxidoreductase (PFO) in anaerobiosis (Catalanotti et al., 2013). In the chloroplast, PFO generates acetyl-CoA and CO_2_ by oxidative decarboxylation of pyruvate and generates reduced ferredoxin (FDX), which can be reoxidized by hydrogenases (HYDA1 and HYDA2), that catalyze proton reduction into H_2_ (Catalanotti et al., 2013). Under anoxic conditions the excess of reduced equivalents may also affect photosynthetic linear electron flow (LEF) by increasing the redox state of pools of electron carriers and electron acceptors such as plastoquinones (PQ), FDX and NADPH. In *C. reinhardtii*, this is reflected by a marked decrease of variable chlorophyll (chl) fluorescence under anoxic conditions (Ghysels et al., 2013; Godaux et al., 2013; Clowez et al., 2015). In addition, a low ATP/NADPH ratio in the chloroplast impairs the activity of Calvin-Benson-Basham cycle (CBB) and therefore limits LEF from water to NADPH. In *C. reinhardtii*, two alternative photosynthetic electron flows (AEFs) contribute to restoring the balance of the ATP/NADPH ratio in the chloroplast upon illumination after acclimation to dark-anoxic conditions: the oxygen-sensitive HYDA1 hydrogenase that reoxidizes FDX reduced by photosystem (PS) I (Hemschemeier and Happe, 2011) and the cyclic electron flow (CEF) around PSI which recycles electrons from PSI acceptor side to intersystem electron carriers (PQ or cytochrome *b_6_f* complex) (Clowez et al., 2015; Godaux et al., 2015). In *C. reinhardtii*, the absence of hydrogenase maturation factors (HYDG or HYDEF) impairs electron transfer during the first seconds of illumination under anoxic conditions (Ghysels et al., 2013; Godaux et al., 2013). In green plants, PGR5 and PGRL1 are the two main proteins that participate in CEF around PSI (DalCorso et al., 2008). In *C. reinhardtii*, the *pgrl1* mutant showed an enhanced H_2_ photoproduction (Tolleter et al., 2011; Godaux et al., 2015) while the double mutant *pgrl1 hydg* cannot reactivate photosynthesis under anoxic conditions (Godaux et al., 2015).

A previous genomic survey identified that among the eukaryotes with secondary plastids, the diatom *Thalassiosira pseudonana* and the excavate *Euglena gracilis* have the most pronounced anaerobic capabilities (Atteia et al., 2013). In anoxic conditions, the excavate *Euglena gracilis* degrades its reserve polysaccharide, paramylon (β-1,3-glucan) (Iwasaki et al., 2019) into myristyl myristate (wax esters) composed of saturated fatty acids and alcohols with chain lengths of 10-18 (Hoffmeister et al., 2005; Inui et al., 2017). During this process, a mitochondrial anaerobic respiration takes place with complex I reducing rhodoquinone that is reoxidized by fumarate reductase (Nakazawa et al., 2018). Unlike common fermentation products described in *C. reinhardtii*, wax esters are accumulated in cells instead of being excreted (Inui et al., 1982; Catalanotti et al., 2013). In addition to wax esters, *E. gracilis* can produce other fermentative metabolites such as lactate and succinate (Tomita et al., 2016). *E. gracilis* also possesses a mitochondrial pyruvate: NADP^+^ oxidoreductase (PNO), an enzyme close to PFO (Rotte et al., 2001; Nakazawa et al., 2003). In a work in 1963, a hydrogenase activity has also been mentioned in *E. gracilis* without any supporting data though (Hartman and Krasna, 1963).

In some diatoms, it was previously demonstrated that an anaerobic respiration pathway called dissimilatory nitrate reduction to ammonium (DNRA) is active during the first hours under anoxic conditions (Kamp et al., 2011; Catalanotti et al., 2013; Kamp et al., 2013; Kamp et al., 2016). Nitrate accumulated at a high intracellular concentration is reduced into nitrite by Nitrate reductase (NR) in the cytosol and nitrite is reduced in the chloroplast into ammonium by a Nitrite reductase (NIR) using FDX as electron donors. Finally, ammonium is excreted out of the cell rather than being assimilated (Lomas and Glibert, 2000; Kamp et al., 2011; Kamp et al., 2013; Kamp et al., 2015). Based on a genomic data survey in four diatoms (*Thalassiosira pseudonana, Phaeodactylum tricornutum*, *Fragilariopsis cylindrus* and *Pseudo-nitzschia*) it was found that only *T. pseudonana* nuclear genome also codes for PFO, PFL and HYDA1 (Atteia et al., 2013).

In *E. gracilis* and *T. pseudonana*, this raises the question of the role of hydrogenase in photosynthetic electron transport under anoxic conditions. In addition, previous studies indicated that CEF around PSI is very low in oxic condition in diatoms (Bailleul et al., 2015) and in *E. gracilis* (Gain et al., 2021). However, the activity of CEF under anoxic conditions has never been studied. Therefore, in this work, we have measured photosystems and hydrogenase activities of *T. pseudonana* and *E. gracilis* in anoxic conditions.

## 2 Material and methods

### 2.1 Strains and growth conditions

Axenic strain of *Thalassiosira pseudonana* (CCMP 1335) is a gift from Angela Falciatore and Benjamin Bailleul (IBPC, Paris, France). *T. pseudonana* was grown under low photosynthetic photon flux density (PPFD) of 50 μmol photons.m^−2^.s^−1^ [white light-emitting diode (LED)], 12 h light – 12 h dark) at 18°C, in artificial sea water (salinity of 33 g.L^−1^) F/2 liquid medium (Guillard and Ryther, 1962; Guillard, 1975) with silica (Sigma-Aldrich, G9903). Axenic strain of *Euglena gracilis* (SAG 1224-5/25, obtained from the University of Göttingen; Sammlung von Algenkulturen, Germany), and axenic strain of *Chlamydomonas reinhardtii* (C1’ from our collection, derived from 137c strain) were grown under continuous low light (PPFD of 50 μmol photons.m^−2^.s^−1^; white LED) at 25°C in Tris-Acetate-Phosphate (TAP) liquid medium (Hutner et al., 1950; Sueoka, 1960; Gorman and Levine, 1965) supplemented with a mix of vitamins (biotin 10^−7^ %, B12 vitamin 10^−7^ % and B1 vitamin 2×10^−5^ %) in the case of *E. gracilis* (Perez et al., 2014), either on solid (1.5% [w/v] agar (Select Agar, Sigma-Aldrich) or in liquid medium. All cultures were performed under air (*i.e.* 21% atmospheric O_2_). All experiments were conducted with cells harvested in the middle of the exponential phase of growth. Cell concentration was determined by a Beckman Coulter Z2 Counter Analyser (Z2; Beckman, Indianapolis, IN, USA) with a parameter size around 4 µm (*T. pseudonana*), 8 µm (*C. reinhardtii*) and 15 µm (*E. gracilis*).

### 2.2 Methods to achieve anoxic conditions

Liquid cultures were centrifuged (4 min at 4,000 *g* for *T. pseudonana* and 10 min at 1,500 *g* for *E. gracilis*) and cell pellets were resuspended in fresh medium (F/2 for *T. pseudonana* and TAP for *E. gracilis*) at 10^7^ and 10^6^ cells.mL^−1^ for *T. pseudonana* and *E. gracilis*, respectively. Cells were kept in the dark 30 minutes before inducing anoxia. All steps were performed at room temperature (RT, 22 ± 2°C). Erlenmeyer flasks containing cell suspension were transferred in a homemade closed chamber containing less than 0.1 μM of oxygen and bubbled in the dark with nitrogen gas (N_2_). Alternatively, cell suspensions were transferred into sealed 4 mL polystyrene cuvettes in presence of glucose (10 mM, Sigma-Aldrich), glucose oxidase (GOX, 2 mg.mL^−1^, ROTH) and catalase (1000 U.mL^−1^, Sigma-Aldrich). Depletion in O_2_ (time 0) was determined using O_2_ sensor spots and optical fibers from Pyroscience (Aachen, Germany). GOX activity produces gluconolactone and H_2_O_2_ from glucose and O_2_ (Bankar et al., 2009). We found that gluconolactone addition in oxic conditions inhibits photosynthetic activity of *T. pseudonana* (**Supplementary Figure S1**). Therefore, unless specifically specified, the N_2_ method was employed to achieve anoxia in this marine diatom. Oxic condition corresponds to aerated cultures (*i.e.* 21% atmospheric O_2_).

### 2.3 Inhibitors preparation

3-(3,4-dichlorophenyl)-1,1-dimethylurea (DCMU), PSII inhibitor, was used at a final concentration of 10 µM. Stock solution of 1 M DCMU was prepared in DMSO. 2-hydroxyacetaldehyde (glycolaldehyde, GA) was used at a final concentration of 20 mM. GA dimer powder was solubilized in water and heated at 65°C for 10 min to obtain 2 M of monomer (Anderson et al., 2007; Roberty et al., 2014). 3-bromopyruvic acid (3BP), was used at a final concentration of 2 mM for *T. pseudonana* and 10 mM for *E. gracilis*. Stock solution of 1 M 3BP was prepared in water.

### 2.4 *In vivo* chlorophyll fluorescence measurements

*In vivo* chl fluorescence measurements were performed on cell suspension, at room temperature (RT, 22 +/- 5°C), with a JTS-10 spectrophotometer (Biologic) with a 640 nm LED source as actinic light. The effective photochemical yield of PSII (ΦPSII) was calculated as (F_M’_ – F_S_)/F_M’_ and the maximal quantum yield of PSII (F_V_/F_M_) was calculated as (F_M_ – F_0_)/F_M_ , where F_0_ is the fluorescence value of dark-acclimated cells, F_S_ is the fluorescence level in response to a given actinic light, and F_M_ , F_M’_ are the maximum fluorescence level induced by a 150 ms pulse of saturating light (3,500 μmol photons.m^−2^.s^−1^). The relative Electron Transfer Rate (rETR_PSII_, µmol electrons.m^−2^.s^−1^) was calculated by multiplying ΦPSII by PPFD used (Genty et al., 1989). All samples were illuminated only once.

### 2.5 Measurement of P700 oxidation

P700 oxidation was measured on cell suspension in presence of 10% (w/v) ficoll by using a JTS-10 spectrophotometer (Biologic, France) at RT as described in Roberty et al. (2014). The quantum yield of photochemical energy conversion by PSI, was calculated as (P_M′_ – P)/(P_M_ − P_0_) (Klughammer and Schreiber, 2008). P_0_ is the absorption level when P700 is fully reduced, P_M_ is the absorption level when P700 is fully oxidized in the presence of 10 mM DCMU (PSII inhibitor) upon saturating continuous illumination, P_s_ is the absorbance level under continuous illumination, and P_M’_ is the maximal absorption level reached during a saturating light pulse (3,500 µmol photons.m^–2.^s^–1^) on top of the actinic light. The maximal P700 absorbance change was estimated by using the P_M_ value. The relative Electron Transfer Rate (rETR_PSI_, µmol electrons.m^-2^.s^-1^) was calculated by multiplying ΦPSI by PPFD used. All samples were illuminated only once.

### 2.6 H_2_ measurement

H_2_ was measured using a hydrogen microsensor (Unisense, UNISENSE A/S, Denmark). The entire set-up was placed in a plastic tent (Glas-Col 108D XX-1H Glove Bag, Templeton Coal Company Inc.) saturated in N_2_ to maintain anoxia. *C. reinhardtii*, *T. pseudonana* and *E. gracilis* cell suspensions (10^7^, 10^7^ and 10^6^ cells. mL^−1^, respectively) were incubated in anoxia during 16 h, and then transferred into a liquid-phase electrode chamber (DW1/AD, Hansatech Instruments) at RT for H_2_ measurement. For each measurement and after stabilization of the signal in the dark, the slope (linear) was determined after 1 min of illumination (200 μmol photon.m^-2^.s^-1^; RGB LED), during 5 min.

### 2.7 Phylogeny analysis

We used sequences of PGRL1, PGR5 of *E. gracilis, T. pseudonana* and *C. reinhardtii*, and of HYDA1 of *C. reinhardtii* and *T. pseudonana* to retrieve sequences using homology searches by BLAST against sequences of the non-redundant protein sequence database of the NCBI and sequences from other databases (MMETSP and data publicly available). We retrieved the top 2000 sequences with an E-value cut-off lower than 1e^− 10^ and aligned them using MAFFT with the quick alignment settings (Katoh and Standley, 2013). Block selection was then performed using BMGE (Criscuolo and Gribaldo, 2010) with a block size of 3 for PGR5 and PGRL phylogeny and a block size of 4 for HYDA1 and the BLOSUM30 similarity matrix. We generated preliminary trees using Fasttree (Price et al., 2010) and “dereplication” was applied to robustly supported monophyletic clades using TreeTrimmer (Maruyama et al., 2013) to reduce sequence redundancy. Then a second tree was obtained using MAFFT and BMGE with the same settings, and we used this tree to select manually the final set of sequences. Finally, proteins were realigned with MUSCLE (Edgar, 2004), block selection was carried out using BMGE with the same settings as above, and trees were generated using IQ-TREE with 100 bootstrap repetitions with the LG4X model.

### 2.8 Statistical analyses

Experiments were performed with at least two independent biological replicates. Comparisons between two treatments were made using two-tailed *t*-tests, comparisons involving more than two treatments were made using a one-way ANOVA and the variation amongst means in relation to treatments was tested by using two-way ANOVA. All statistical analyses were performed using Microsoft Excel software with a threshold of significance at 0.95 (*p* < 0.05).

## 3 Results

### 3.1 Distinct chlorophyll fluorescence signatures in anoxic conditions in *E. gracilis* and *T. pseudonana*

**Figure 1:**
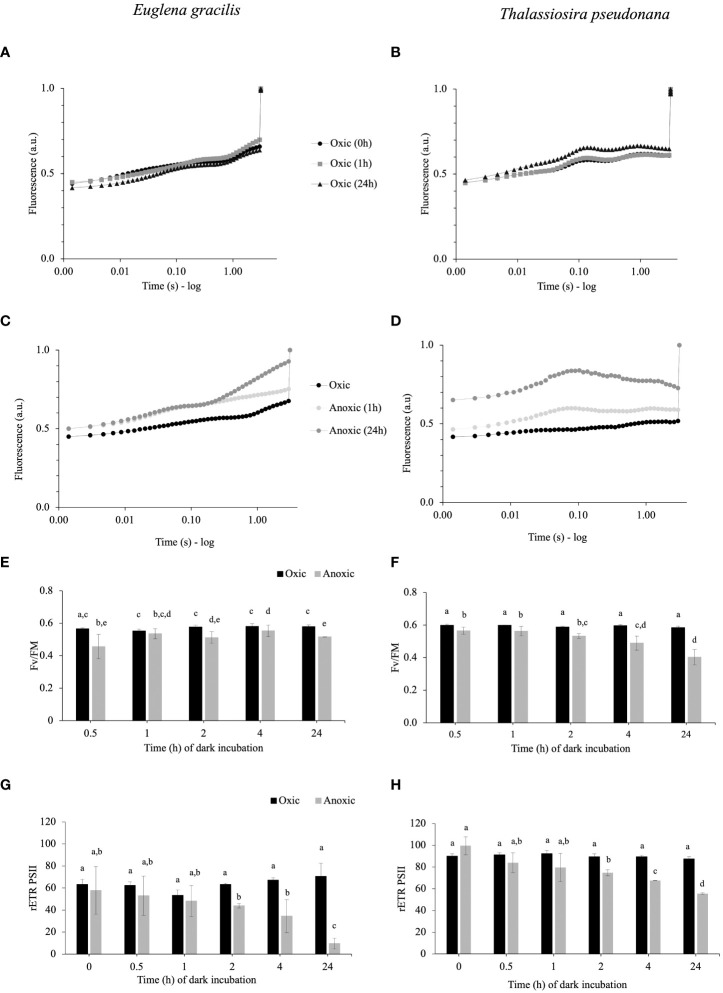
**(A–D)**. Representative chlorophyll fluorescence kinetics obtained with 3 seconds illumination (185 µmol photons.m^-2^.s^-1^) after 1 h and 24 h of incubation in dark oxic **(A, B)** or dark anoxic **(C, D)** conditions in *E gracilis* **(A, C)** and in *T. pseudonana* **(B, D)**. Maximal fluorescence values (F_M_) were normalized to 1. **(E–H)**. Photosynthetic parameters in function of the incubation times in anoxic (grey) or oxic (black) conditions for *E gracilis* and *T. pseudonana*: F_V_/F_M_**(E, F)** and rETR_PSII_ measured after 3 s of illumination at 185 µmol photons m^–2^ s^–1^ **(G, H)**. For *E gracilis*, anoxia was induced by the GOX method. For *T. pseudonana*, anoxia was induced by N_2_ bubbling except for T0 for rETR_PSII_ [**(H)** GOX]. Data are presented as mean ± standard deviation (SD). All measurements were performed for three biological replicates (n=3). Vertical bars indicate the SD and different letters represent the statistical differences between conditions (Anova I, *p* < 0.05).

We compared chl *a* fluorescence kinetics (3 seconds of illumination at subsaturating light) of *E. gracilis* and *T. pseudonana* cells during dark acclimation under oxic or anoxic conditions. In oxic conditions, no change in fluorescence curves, or significant differences in maximal quantum efficiency of PSII (F_V_/F_M_) (**Figures 1A, B**), and relative electron transfer rate of PSII (rETR_PSII_) values could be observed over time in the dark for both species (**Figures 1E–H**). In *E. gracilis*, F_V_/F_M_ in anoxia is always slightly reduced (**Figure 1E**) while rETR_PSII_ is decreased by 31% from 2 h of anoxia and up to 86% after 24 h (**Figure 1G**). After 24 h, this is accompanied by a progressive increase of the fluorescence signal during the first seconds of illumination (**Figure 1C**), indicating a progressive limitation in the availability of electron acceptors. In *T. pseudonana* in anoxic conditions, there is a progressive decrease of F_V_/F_M_ (from 0.6 in control conditions to *ca.* 0.4 after 24 h in anoxia) (**Figure 1F**). The rETR_PSII_ after 3 s also progressively significantly decreases over time in anoxic conditions (by *ca.* 40% after 24 h) (**Figure 1H**). After 24 h, this is accompanied by a peculiar signature in the fluorescence curves: in a few ms, the fluorescence signal reaches a maximal transient value which is close to F_M_ and then it decreases (**Figure 1D**).

### 3.2 3-Bromopyruvate impairs PSII activity at the onset of light under anoxic conditions

**Figure 2:**
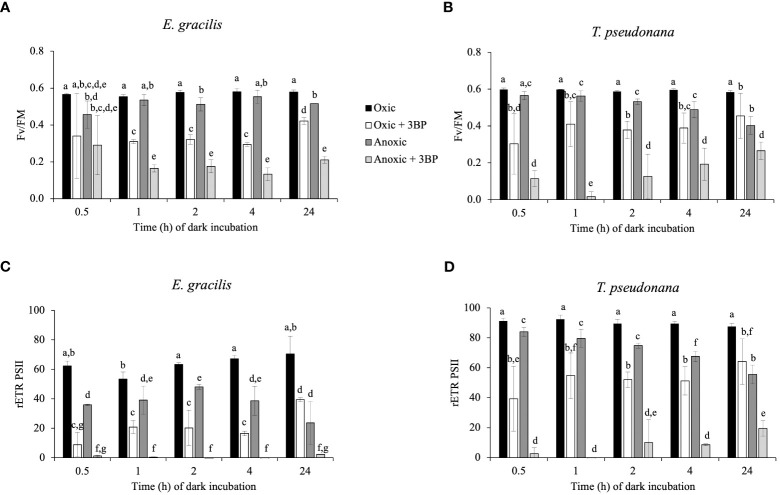
Impact of 3-bromopyruvic acid (3BP, 10 mM for *E. gracilis* and 2 mM for *T. pseudonana*) on F_V_/F_M_ **(A, B),** and rETR_PSII_ (3s illumination at 185 μmol photons.m^-2^.s^-1^, **C, D**) in function of dark incubation under oxic (control, black and white) or anoxic (grey) conditions on *E. gracilis* **(A, C)** and *T. pseudonana* **(B, D)**. Data are presented as mean ± SD. All measurements were performed for three biological replicates (n=3). 3BP (anoxic solution) was added 10 min before illumination. Different letters represent the statistical differences between conditions (Anova II, *p* < 0.05).

After dark anoxic incubation, the occurrence of significant rETR_PSII_ after 3 s of illumination in *E. gracilis* (**Figures 1C, G**) and *T. pseudonana* (**Figures 1D, H**) reflects the availability of oxidized photosynthetic electron acceptors, and therefore the activity of at least one pathway able to reoxidize photosynthetic electron acceptors in anoxic conditions. To determine if the availability of oxidized photosynthetic electron acceptors during the onset of light depends on a catabolic pathway, we tested the effect of 3-bromopyruvic acid (3BP). 3BP has a large range of possible targets pertaining to the catabolism (Honer Zu Bentrup et al., 1999; Shoshan, 2012; Pedersen, 2012; Sprowl-Tanio et al., 2016). We selected 3BP concentrations (2 mM for *T. pseudonana* and 10 mM for *E. gracilis*) that inhibit after 10 min about 80% of the dark oxygen consumption rate by the mitochondrial respiration in oxic conditions (**Supplementary Figure S2**). In both species, addition of 3BP under anoxic conditions 10 min before illumination almost fully abolished F_V_/F_M_ and rETR_PSII_ (at least by 80%) while it had a lesser impact on rETR_PSII_ and F_V_/F_M_ in oxic conditions (**Figure 2**). These results suggest that in both species resuming photosynthetic chain activity is more dependent on catabolism under anoxic conditions than in oxic conditions in both *T. pseudonana* and *E. gracilis*.

### 3.3 No evidence for a sustained hydrogenase activity in anoxic conditions in *T. pseudonana* and *E. gracilis*

**Figure 3:**
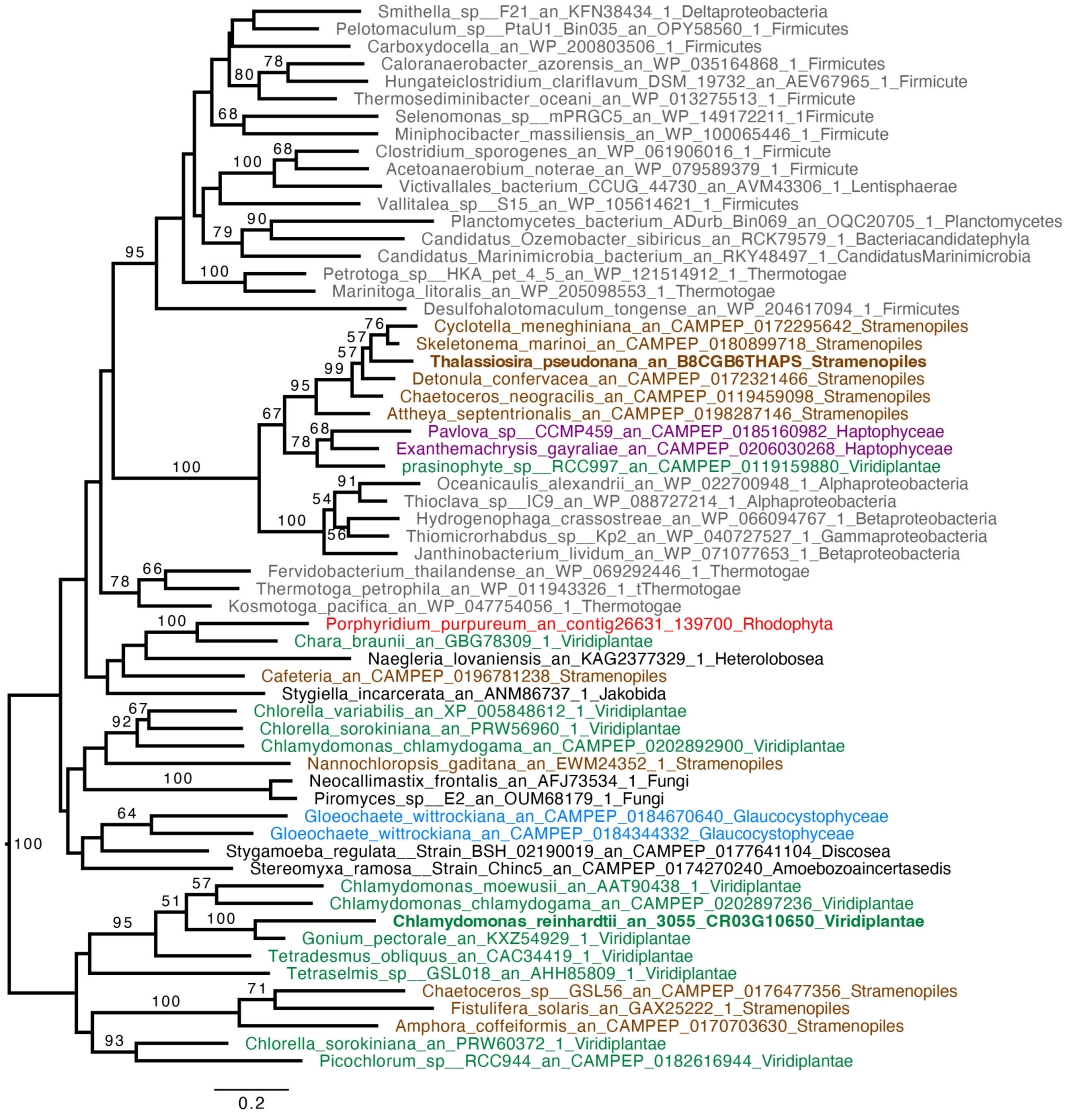
Hydrogenase (HYDA1) phylogenetic tree. The tree is midpoint rooted and represents the tree obtained with maximum likelihood approach. We used IQTREE under the LG4X model and performed bootstraps analysis with 100 bootstraps repetition. Bootstrap values > 50% are mapped into the nodes. The scale bar shows the inferred number of amino acid substitutions per site. Sequences are highlighted in grey for Bacteria, in brown for Stramenopila, purple for Haptophyta, light green for Viridiplantae, red for Rhodophyceae, light blue for Glaucophyta while other eukaryotes are in black. Sequences of *C. reinhardtii* and *T. pseudonana* are in bold.

**Figure 4:**
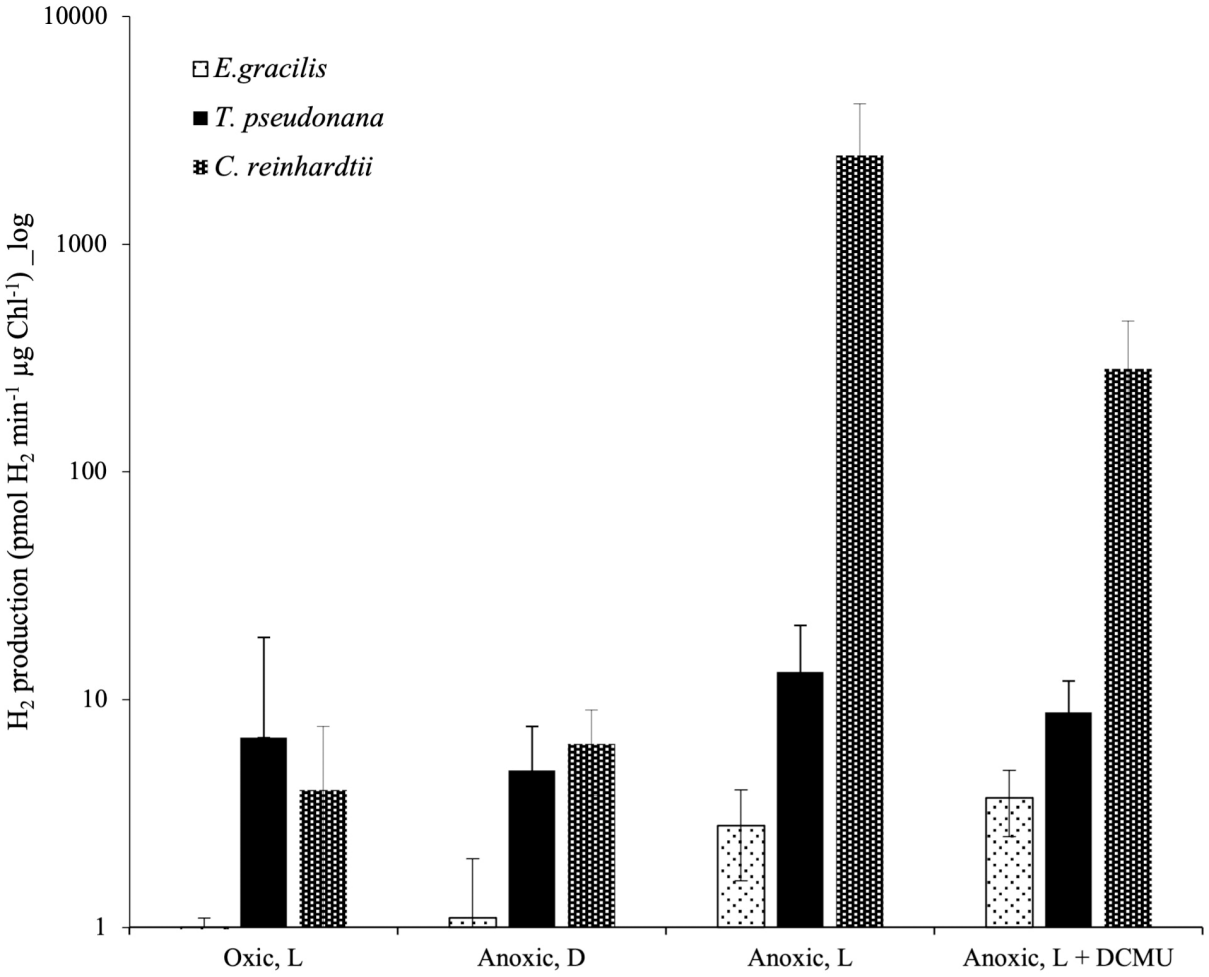
*In vivo* hydrogen evolution rate in *E. gracilis, T. pseudonana* and *C. reinhardtii* in oxic and anoxic conditions. Data are presented as mean ± SD. All measurements were performed on at least three biological replicates. Vertical bars indicate the SD. L, Actinic Light; D, Dark; DCMU was added at 10 µM.

*C. reinhardtii* rETR_PSII_ at the onset of light under anoxic conditions depends on the activity of an oxygen-sensitive hydrogenase (HYDA1) that oxidizes FDX, the acceptor of PSI (Godaux et al., 2013; Godaux et al., 2015). When turning our attention to hydrogenase phylogeny, no gene coding related to *C. reinhardtii* HYDA1 hydrogenase could be identified in *E. gracilis*. A search performed with the sequences of other hydrogenases (Ni-Fe and Fe-only hydrogenases*, from Hydrogenovibrio marinus (KDN94743.1) and C. reinhardtii* (AAR04931.1), respectively) also yielded no result. The only sequence found was a protein closely related to the NAR (nuclear architecture related) protein family (EG_transcript_7200). In contrast, a sequence related to *C. reinhardtii* hydrogenase HYDA1 is found in *T. pseudonana* (B8CGB6_THAPS) and in a limited number of other Coscinodiscophyceae representatives such as *Cyclotella meneghiniana* and *Skeletonema marinoi* while grouping strongly with high bootstraps value (BS=100) with different bacteria (Proteobacteria). We were however unable to retrieve clear, expected, taxonomic groups (**Figure 3**), questioning vertical evolution of mechanisms linked to hydrogen. In both *E. gracilis* and *T. pseudonana* cells maintained in anoxic conditions, max H_2_ evolution rates were around 5 pmol min^-1^ µg chl^-1^ and they did not increase upon subsequent illumination under anoxic conditions. These values were not different from the values measured in dark oxic conditions (**Figure 4**). In *C. reinhardtii*, used as a control, H_2_ evolution rate was also very low in dark anoxic or oxic conditions (*ca.* 6 pmol min^-1^ µg chl^-1^). However, in *C. reinhardtii* in anoxic conditions, as expected (Godaux et al., 2013), it strongly increased during subsequent illumination, at about 2.5 nmol min^-1^ µg chl^-1^ and almost 90% of this H_2_ evolution rate in the light was inhibited in the presence of DCMU (**Figure 4**).

### 3.4 The increase in PSII activity depends on the reactivation of CBB under anoxic conditions

**Figure 5:**
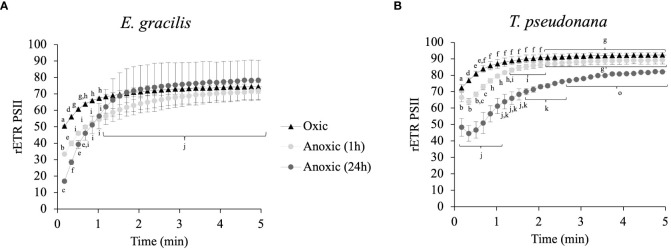
Relative electron transfer rate (rETR_PSII_) kinetics during continuous illumination (185 µmol photons.m^–2^.s^–1^) for different anoxic incubation times, compared to oxic control (dark adapted for 1 h) in *E gracilis* **(A)** and *T. pseudonana* **(B)**. Data are presented as mean ± SD; at least two biological replicates. Different letters represent the statistical differences between conditions (Anova I, *p* < 0.05).

In parallel to measurement of *in vivo* H_2_ evolution in continuous light, we monitored rETR_PSII_. In *E. gracilis* and in *T. pseudonana* we observed a progressive increase of rETR_PSII_ which saturates at similar values after several minutes of illumination in both oxic and anoxic conditions (**Figure 5**). In *E. gracilis*, despite the low initial rETR_PSII_ in anoxia, the kinetics of reactivation are similar to the one in oxic condition (*i.e.* half-maximum rate is achieved after 30-60 s) (**Figure 5A**). In *T*. *pseudonana*, there is a short lag (< 30 s) before rETR_PSII_ increases in anoxia (**Figure 5B**). After 24 h in anoxia, this delay is associated with a slower reactivation compared to the oxic conditions (**Figure 5B**).

**Figure 6:**
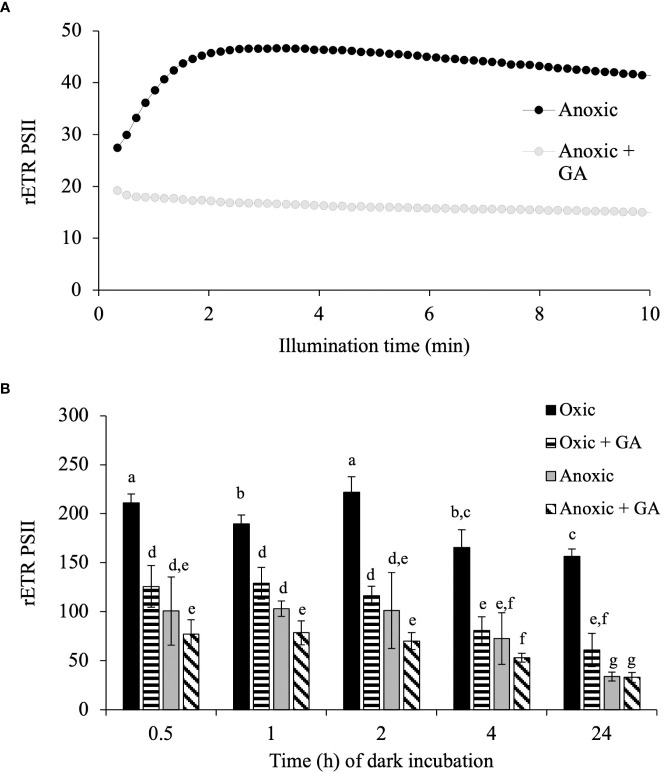
Impact of glycolaldehyde in *T. pseudonana*. **(A)** relative Electron Transfer Rate (rETR) of PSII, μmol electron. m^-2^. s^-1^) in *T. pseudonana* during continuous illumination (185 μmol photons. m^-2^. s^-1^) after 1 h of anoxic incubation. 20 mM glycolaldehyde (+ GA) was added 10 min prior illumination. **(B)** Impact of glycolaldehyde (GA, 20 mM) on rETR_PSII_ (3s illumination at 885 µmol photons.m^-2^. s^-1^) in function of incubation time in the dark in oxic (control) or anoxia on *T. pseudonana*. Data are presented as mean ± SD. All data were performed for three biological replicates. Different letters represent the statistical differences between conditions in function of time of dark incubation (Anova II, *p* < 0.05).

In *C. reinhardtii*, such an increase of rETR_PSII_ is due to the reactivation of the Calvin-Benson-Bassham (CBB) cycle (Cournac et al., 2002; Godaux et al., 2015). To determine if it is also the case for *E. gracilis and T. pseudonan*a, we first tested the effect of different concentrations of glycolaldehyde (GA), reported to inhibit the last enzymatic step of the CBB cycle (phosphoribulokinase), on photosynthetic electron transfer capacity in oxic conditions. In *E. gracilis*, despite phosphoribulokinase is present (Petersen et al., 2006), addition of GA (up to 100 mM) had no effect on photosynthesis in oxic conditions (**Supplementary Figure S3A**) and was therefore not tested in anoxic conditions. In *T. pseudonana*, the higher is the GA concentration, the higher is the inhibition of photosynthetic activity (**Supplementary Figure S3B**). On light-acclimated cells in oxic conditions, the addition of GA (20 mM) led to inhibition of 24% of rETR_PSII_ under low light (185 µmol photons.m^-2^. s^-1^) and up to 72% of rETR_PSII_ under high light (2840 µmol photons.m^–2^.s^–1^) (**Supplementary Figure S3C**). Addition of 20 mM GA was then added on *T. pseudonana* cells acclimated during 24 h to anoxic conditions in the dark and it fully prevented the increase of rETR_PSII_ during 10 min continuous illumination in anoxic conditions (**Figure 6A**). GA addition also partly inhibited rETR_PSII_ after 3 s of illumination in oxic conditions, while it had a lesser effect, or no effect on the rETR_PSII_ after 3 s of illumination in anoxic conditions (**Figure 6B**).

### 3.5 PSI is more active than PSII at the onset of light in anoxic conditions

**Figure 7:**
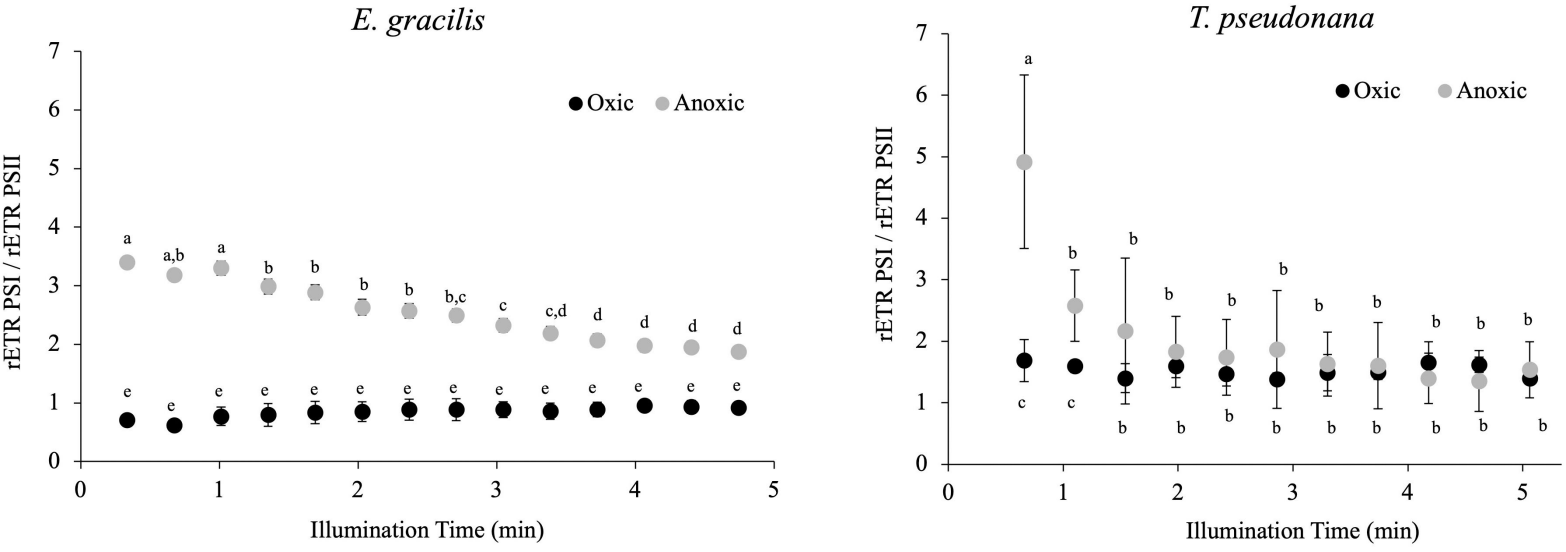
Change in the ratio between rETR_PSI_ and rETR_PSII_ during continuous illumination (185 µmol.photons.m^–2^.s^–1^) after 24 h in the dark under anoxic or oxic conditions (control) in *E. gracilis* and *T. pseudonana*. Data are presented as mean ± SD. All measurements were performed for three biological independent culture replicates (n = 3). Different letters represent the statistical differences between conditions (ANOVA I, *p* < 0.05).

**Figure 8:**
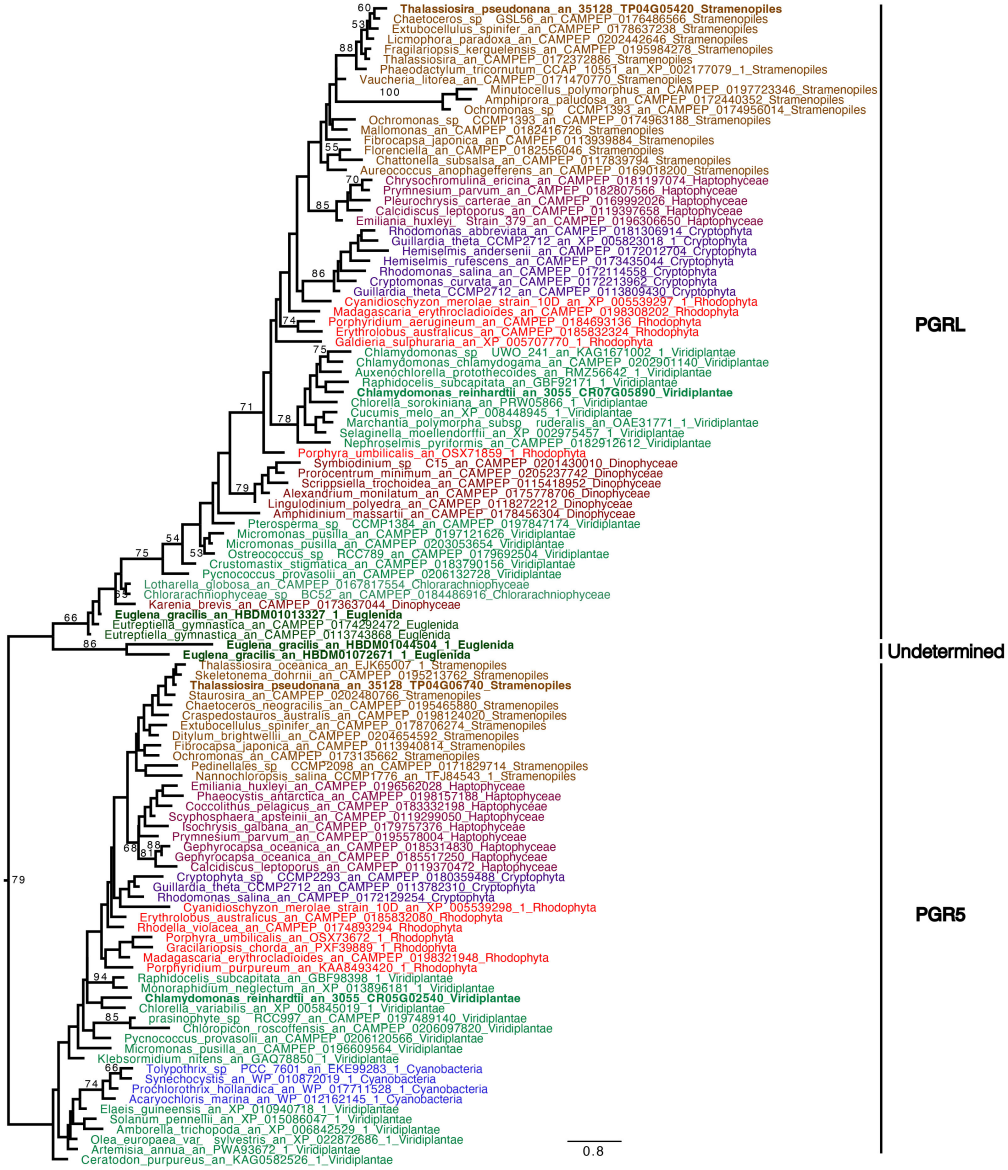
Phylogenetic tree of PGR5 and PGRL1. The tree displayed is midpoint rooted and represents the tree obtained with maximum likelihood approach. We used IQTREE under the LG4X model as well as performing bootstraps analysis with 100 bootstraps repetition. Bootstrap values >50% are mapped into the nodes. The scale bar shows the inferred number of amino acid substitutions per site. Sequences are highlighted in blue for Cyanobacteria, in light brown for Stramenopila, and dark brown for Alveolata, purple for Cryptista, garnet for Haptophyta, light green for Viridiplantae, dark green for Euglenozoa and turquoise blue for Chlorarachniophytes, red for Rhodophyceae. Sequences of *C. reinhardtii*, *E. gracilis* and *T. pseudonana* are in bold.

In *C. reinhardtii*, the reactivation of the CBB cycle from dark anoxic conditions requires CEF around PSI in addition to hydrogenase activity (Godaux et al., 2015). To determine if CEF around PSI occurs in anoxic conditions in *E. gracilis* and *T. pseudonana*, we compared the values of rETR_PSI_ and rETR_PSII_ in oxic and after 24h of anoxic conditions (**Figure 7**). After 30 s of illumination, the ratio between rETR_PSI_ and rETR_PSII_ is higher in anoxic conditions (3.4 ± 0.1 for *E. gracilis* and 4.9 ± 1.4 for *T. pseudonana*) than in oxic conditions (0.9 ± 0.1 for *E. gracilis* and 1.5 ± 0.1 for *T. pseudonana*). After 5 min of illumination, this ratio significantly decreased in anoxic conditions in both species (1.8 ± 0.1 for *E. gracilis* and 1.5 ± 0.5 for *T. pseudonana*) while it remained stable in control conditions. These values suggest that in both species, CEF around PSI is active in anoxic conditions. In green plants, the main proteins involved in CEF are PGRL1 and PGR5 (DalCorso et al, 2008). We conducted a phylogenetic analysis to identify putative PGRL1 and PGR5 orthologous genes in *E. gracilis* and in *T. pseudonana*. (**Figure 8**). The presence of *E. gracilis* (HBDM01013327), *T. pseudonana* (TP04G05420), and *C. reinhardtii* (CR07G05890) indicates a conservation of PGRL1 in those lineages. We also observe in *T. pseudonana* (TP04G06740) an orthologous gene of *C. reinhardtii* PGR5 (CR05G02540) while no clear PGR5 could be retrieved in *E. gracilis*.

## 4 Discussion

In their natural environment, some microalgae can be exposed to hypoxic or anoxic environments. In the model green alga *C. reinhardtii*, light-dependent oxygen-sensitive hydrogenase activity, and cyclic electron flow around photosystem I, both contribute to restoring photosynthetic linear electron flow in anoxic conditions (Godaux et al., 2013; Godaux et al., 2015). In this work, we analyzed photosynthetic electron transfer after incubation in dark anoxic conditions in *T. pseudonana* and *E. gracilis*, two model microalgae species that possess canonical fermentative enzymes (Atteia et al., 2013), but also peculiar fermentative pathways (Nakazawa et al., 2003; Kamp et al., 2011; Kamp et al., 2015; Iwasaki et al., 2019). In both species, PSII activity measured as its relative electron transfer rate (rETR_PSII_) was low at the onset of light after acclimation in the dark under anoxic conditions, but increased progressively (**Figure 5**), reflecting an increase of photosynthetic LEF. In the primary green alga *C. reinhardtii* increase of LEF in these conditions depends on the reactivation of the CBB cycle (Cournac et al., 2002; Ghysels et al., 2013; Godaux et al., 2015). In green plants, addition of glycolaldehyde (GA), inhibits phosphoribulokinase, an essential enzyme of the CBB cycle, and therefore the photosynthetic electron transport rate (Sicher, 1984; Takahashi and Murata, 2005). In *T. pseudonana* after 24h in anoxic conditions, addition of GA prevents the increase of PSII activity upon continuous illumination conditions (**Figure 6A**). Despite GA may have side effects on other enzymes (see e.g. Jayakody et al., 2011) these results strongly suggests that increase of PSII activity is due to resuming of CBB activity in *T. pseudonana*. Kinetics of activation in both *T. pseudonana* and *E. gracilis* are similar to those observed in *C. reinhardtii* (Takahashi and Murata, 2005; Godaux et al., 2015) and in the cyanobacteria Aphanocapsa (Pelroy et al., 1976). In this respect, the significant inhibitory effect of GA on PSII activity after 3 s of illumination in oxic conditions in *T. pseudonana* (**Figure 6B**) and the absence of lag in PSII activity increase in oxic conditions in both *T. pseudonana* and *E. gracilis* (**Figure 5**) reinforces the idea that CBB cycle is activated very fast in these microalgal species. In contrast, the minor effect, or the absence of effect of GA on the initial activity of PSII in anoxia in *T. pseudonana* (3s, **Figure 6B**) suggest that the initial PSII activity (3s) is independent of the CBB cycle activity in anoxia and therefore relies on another electron sink.

### 4.1 Cyclic electron flow around PSI is active in anoxia and may contribute to resuming photosynthetic activity

In *C. reinhardtii*, two alternative electron pathways, namely HYDA1, a Fe-Fe hydrogenase that accepts electrons at the acceptor side of PSI, and CEF around PSI, are also required to resume photosynthesis (Godaux et al., 2015). In *E. gracilis* and *T. pseudonana*, rETR_PSI_ is much higher than rETR_PSII_ at onset of light under anoxic conditions (**Figure 7**). In green plants, including *C. reinhardtii*, this observation is usually interpreted as a large fraction of PSI operating independently of PSII, and therefore contributing to CEF (Godaux et al., 2015; Fan et al., 2016). Our results therefore indicate that CEF around PSI is large but transient under anoxic conditions, whereas in comparison it could be very weak in oxic conditions in both *T. pseudonana* and *E. gracilis*. This is in line with our previous studies indicating that CEF around PSI is very low in oxic condition in diatoms (Bailleul et al., 2015) and in *E. gracilis* (Gain et al., 2021). CEF is also higher in anoxic conditions than in oxic conditions in *C. reinhardtii* (Alric, 2010), suggesting that the mechanisms behind LEF and CEF partitioning may be broadly shared in photosynthetic eukaryotes. In this respect, the main proteins involved in CEF in green plants, PGRL1 and PGR5 (DalCorso et al, 2008), have orthologs in *T. pseudonana* and at least PGRL1 has an ortholog in *E. gracilis* (**Figure 8**). In addition, in *E. gracilis* two gene sequences (HBDM01044504, HBDM01072671) are related to PGR proteins (**Figure 8**).

### 4.2 Very low hydrogenase activities in *T. pseudonana* and *E. gracilis*

Based on a previous genomic survey, a gene coding for a hydrogenase was identified in *T. pseudonana* (Atteia et al., 2013). It belongs to the same [FeFe] hydrogenase family that is present in green algae such as *C. reinhardtii* (**Figure 3**). We show here that this enzyme is shared only by Coscinodiscophyceae among diatoms. This points to an independent lateral gene transfer (LGT) between specific diatoms and bacteria, but the direction is unclear (**Figure 3**). From a functional point of view, this could, in case of LGT from bacteria to diatoms, correlate with special activities or different use in *T. pseudonana* compared to *C. reinhardtii*. In this respect, HYDEF and HYDG, two maturation factors required for HYDA1 maturation in *C. reinhardtii* have not been found in the genome of *T. pseudonana* (Atteia et al., 2013), further supporting the idea that the *T. pseudonana* [FeFe] hydrogenase does not share a common role with *C. reinhardtii* HYDA1. In *E. gracilis*, no HYDA hydrogenase could be identified but only a protein related to the NAR protein family. NAR protein family most probably evolved from an ancestral Fe-hydrogenase and are known to not produce hydrogen (Hackstein, 2015). H_2_ evolution was barely detectable in both *T. pseudonana* and *E. gracilis*. These values were also not significantly different between oxic and anoxic conditions, and more specifically not stimulated by light in anoxic conditions (**Figure 4**). The maximal H_2_ evolution rates *in vivo* measured here for *C. reinhardtii* are in good agreement with previous values measured in subsaturating light (0.25 to 0.58 nmol H_2_.min^-1^.µg chl^-1^) (Tolleter et al., 2011; Clowez et al., 2015; Godaux et al., 2015) or in saturating light (*ca* 2 nmol H_2_.min^-1^.µg chl^-1^) (Meuser et al., 2012; Godaux et al., 2013). Overall, this makes the role of a hydrogenase putative activity in *T. pseudonana* and *E. gracilis* in photosynthetic electron transfer very unlikely. In this respect, *C. reinhardtii*, hydrogenase-deficient mutants impaired in HYDG or HYDEF maturation factors of HYDA1 are however still able to resume photosynthesis provided that CEF around PSI operates (Ghysels et al., 2013; Godaux et al., 2015). In *C. reinhardtii*, several oxidases directly connected to the photosynthetic electron transfer chain (e.g. Flavodiiron protein or Plastidial Terminal Oxidase) have also been described as electron valves for photosynthetic electrons when O_2_ released by PSII becomes available (Cardol et al., 2010; Godaux et al., 2015; Burlacot et al., 2018). Still, *C. reinhardtii* oxidases do not contribute to the very initial PSII activity at the onset of light (Godaux et al., 2013). Indeed, as in other green algae (Kessler, 1973; Schreiber and Vidaver, 1974), PSII activity during the first seconds of illumination after an anoxic incubation in the dark strictly depends only on *HYDA1* expression (Forestier et al., 2003; Godaux et al., 2013). This strongly suggests that in *T. pseudonana* and *E. gracilis* at least one O_2_-independant alternative electron sink is also active under anoxic conditions and contributes to the observed LEF at the onset of light (**Figure 5**).

### 4.3 Candidate alternative photosynthetic electron sink under anoxic conditions

At variance with *C. reinhardtii* (Godaux et al., 2013; Clowez et al., 2015), a residual rETR_PSII_ under anoxic conditions can be measured as soon as oxygen is depleted (time “0” in **Figures 1G, H**). This result agrees with previous results obtained in *E. gracilis* and in *P. tricornutum*, another diatom (Shimakawa et al., 2017), and suggests that the electron sink is already present in oxic conditions. In both *T. pseudonana* and *E. gracilis*, the decrease of rETR_PSII_ capacity at light onset over time under anoxic conditions in the dark (**Figures 1G, H**) suggests that the capacity of the unknown electron sink(s) decreased. In *T. pseudonana*, this is accompanied by a progressive decrease of Fv/FM (**Figures 1D, F**), suggesting that the PSII acceptor pool (Q_A_, PQ) is more reduced, probably due to an increase of reducing power in the stroma. In addition, PSII activity at light onset is abolished in presence of 3BP to a larger extent in anoxic conditions in both species (**Figure 2**). 3BP is not a specific inhibitor and may inhibit the glyceraldehyde-3-phosphate dehydrogenase, the hexokinase II, isocitrate lyase, some monocarboxylate transporters and directly or indirectly some respiratory enzymes in other species (Honer Zu Bentrup et al., 1999; Shoshan, 2012; Pedersen, 2012, Sprowl-Tanio et al., 2016). Thus, our results suggest that the capacity of this initial photosynthetic electron sink in anoxic condition may depend on a catabolic pathway, and maybe be related to glycolysis and/or some fermentative pathways.

Under anoxic conditions, *E. gracilis* degrades paramylon, a β-1,3-glucan-type polysaccharide (Barsanti et al., 2001), into wax-ester (Inui et al., 1982; Yamada et al., 2019). This pathway is active when anoxia is reached because wax esters are already detected after 5 min in anoxic conditions (Inui et al., 1982), and it involves a mitochondrial anaerobic respiration (Nakazawa et al., 2018). In anoxic conditions, reducing equivalents are shuttled from cytosol into mitochondria to generate mainly NADPH (Nakazawa et al., 2021). Therefore, in *E. gracilis* we hypothesize that the export of reducing power out of chloroplasts into mitochondria occurs under anoxic conditions and contributes to the observed electron sink at light onset to prevent over-reduction of the photosynthetic apparatus. In *Thalassiosira pseudonana*, nitrate reduction to ammonium by the ferredoxin-NIR (NIR1) activity of the DNRA pathway (Kamp et al., 2011; Kamp et al., 2013) has all the characteristics to correspond to the initial photosynthetic electron sink identified in this work under anoxic conditions, i.e. part of the electrons released at PSII may be used for nitrate reduction during dark/anoxic-to-light transition. Ammonium is excreted out of the cell rather than being assimilated (Lomas and Glibert, 2000; Kamp et al., 2011; Kamp et al., 2013; Kamp et al., 2015). In *Thalassiosira weissflogii* and *Amphora coffeaeformis*, nitrate concentration decreases by half after 2 h and 6 h, respectively, under anoxic conditions (Kamp et al., 2011; Kamp et al., 2013). DNRA is also already active in oxic conditions where this pathway acts as a photosynthetic electron sink to prevent an over-reduction of the photosynthetic apparatus in high light conditions or during an irradiance shift (Lomas and Glibert, 2000; Lomas et al., 2000). In that respect, the rate of NH_4_^+^ release in *T. weissflogii* in the light reaches 150 fmol NH_4_^+^ h^-1^ cell^-1^ at 20°C (Lomas et al., 2000), *i.e.* 0.5 nmol NH_4_^+^ min^-1^ µg chl^-1^ assuming a chl content of 5 pg per cell (Walter et al., 2015), a value of the same magnitude as the rate of oxygen evolution (*ca.* 4 nmol O_2_ min^-1^ µg chl^-1^) reported for *T. weissflogii* in oxic conditions (Goldman et al., 2017).

## 5 Concluding remarks

In conclusion we have observed in *T. pseudonana* and *E. gracilis* that resuming photosynthetic activity under anoxic conditions is possible even in absence of an active hydrogenase. This suggests the presence of anaerobic alternative electron flow with a similar role than *C. reinhardtii* hydrogenase, which helps to prevent an over reduction of the photosynthetic apparatus and optimizes the CBB reactivation at the onset of light. Further phylogenetic analyses are required to fully understand the diversity and origins of these pathways. In addition, the characterization of the spectrum of fermentation products of these complex algae as well as of photosynthesis regulation under these conditions are still poorly studied compared to other model algae. The study of these organisms may lead to surprising discoveries like original anaerobic pathways (*i.e*. DNRA, wax esters) which can be important for the physiology and the ecology of these algae, but also from a biotechnological point of view with the production of valuable compounds (e.g. wax esters).

## Data availability statement

The raw data supporting the conclusions of this article will be made available by the authors, without undue reservation.

## Author contributions

PC directed the research. GG, NB, UC, DG and PC designed experiments. GG, TF and NB performed biophysical experiments and physiological analyses. GG, TF, and DG measured hydrogen production. UC performed phylogenetic trees. GG, NB, UC, TF, and PC analyzed and interpreted data. GG, UC, and PC generated all figures and supporting data. GG and PC wrote the manuscript with comments of all authors. All authors contributed to the article and approved the submitted version.
