# Supplementary material for: Induction of photosynthesis under anoxic condition in Thalassiosira pseudonana and Euglena gracilis: interactions between fermentation and photosynthesis
Source: Front Plant Sci. 2023 Jul 25;14:1186926. doi: 10.3389/fpls.2023.1186926 (PMC10407231; doi:10.3389/fpls.2023.1186926)
Supplement: Supplementary file 1 [file DataSheet_1.docx]

**Fig. S1 - Impact of the anoxia induction method on *T. pseudonana* and *E. gracilis.*** The photosynthetic activity of *E. gracilis* and *T. pseudonana* was determined after 0 and 1 h of incubation under dark-anoxic conditions by illuminating the cell suspension for 3 s under a moderate actinic light (185 μmol photons.m^−2^.s^−1^), a light that gives rise to ~ 20 % of maximal electron transfer rate in oxic conditions by using GOX method. The rETR_PSII_ of *E. gracilis* decreased with anoxia time but remained not null (**Figure S1**) while in *T. pseudonana*, after a few hours of incubation by GOX method, the rETR_PSII_ was null (data not shown). As the GOX method led to the production of gluconolactone, a glucose derivative (Bankar *et al.,* 2009), we investigated if gluconolactone did not impact photosynthesis on oxic conditions (O_2_). By testing the effect of the gluconolactone in oxic conditions on *T. pseudonana*, we observed that a rETR_PSII_ completely inhibited after one hour of incubation in presence of 10 mM of gluconolactone, unlike *E. gracilis* (**Figure S1**). As a result, we conclude that gluconolactone is responsible for the phenotype observed in anoxia. These observations indicate that gluconolactone inhibits photosynthesis in *T. pseudonana* regardless of oxygen availability. However, since gluconolactone has no effect during the first min, we assume that the first rETR measurements obtained with the GOX method reflect the capacity of electron sinks in the sudden absence of oxygen (time 0).

**Figure S1.** relative Electron Transfer Rate (rETR) obtained after 3 s of illumination (185 µmol .photons.m^-2^.s^-1^) after 0 and 1 h of dark-anoxic incubation (grey) compared to oxic conditions (control, black) and to oxic conditions in presence of gluconolactone (10 mM, white) in *E. gracilis* and in *T. pseudonana*. The gluconolactone concentration corresponds to the substrate (glucose) concentration added into the sample. Data are presented as mean ± standard deviation (SD). All measurements were performed for three biological independent culture replicates (n=3). Different letters represent the statistical differences between conditions (Anova II, *p* < 0.05).

**Fig. S2 - Impact of 3-bromopyruvic acid on dark-oxygen consumption rate**

**Figure S2.** A. Impact of 3-bromopyruvic acid (10 mM for *E. gracilis (white) and 2 mM for T. pseudonana (striped*) on the dark oxygen consumption rate (VO_2_) by the mitochondrial respiration in oxic conditions (control) after 0, 5 and 10 minutes of incubation. All values were normalized on the value of dark oxygen consumption rate at time 0. B. Impact of different concentrations of 3-Bromopyruvate (mM) on E. gracilis (white) and T. pseudonana (striped) on respiration rate. Data are presented as mean ± standard deviation (SD). All measurements were performed for three biological independent culture replicates. Different letters represent the statistical differences between conditions (Anova I, *p* < 0.05)

**Fig. S3 - Impact of glycolaldehyde on rETR_PSII_ in light acclimated cells of *E. gracilis* and *T. pseudonana.***

**Figure S3.** Effect of various concentrations of glycolaldehyde (GA) on the rETR_PSII_ (μmol electron.m^-2^.s^-1^) in light acclimated cells of *E. gracilis* (**A**) and *T. pseudonana* (**B**), normalized on the values without inhibitor. Values obtained after 5 min of illumination (185 μmol photons.m^–2^. s^–1^). **C.** Effect of glycolaldehyde (GA, 20 mM) on rETR_PSII_ at different PPFD (µmol.photons.m^-2^.s^-1^; 5 min of illumination) in light-adapted *T. pseudonana* cells (oxic conditions). Data are presented as mean ± standard deviation (SD). All measurements (**A, B, C**) were performed for three biological independent culture replicates (n=3). Different letters represent the statistical differences between conditions (Anova I, *p* < 0.05).
