# Supplementary figures and images for: Induction of photosynthesis under anoxic condition in Thalassiosira pseudonana and Euglena gracilis: interactions between fermentation and photosynthesis
Source: Front Plant Sci. 2023 Jul 25;14:1186926. doi: 10.3389/fpls.2023.1186926 (PMC10407231; doi:10.3389/fpls.2023.1186926)

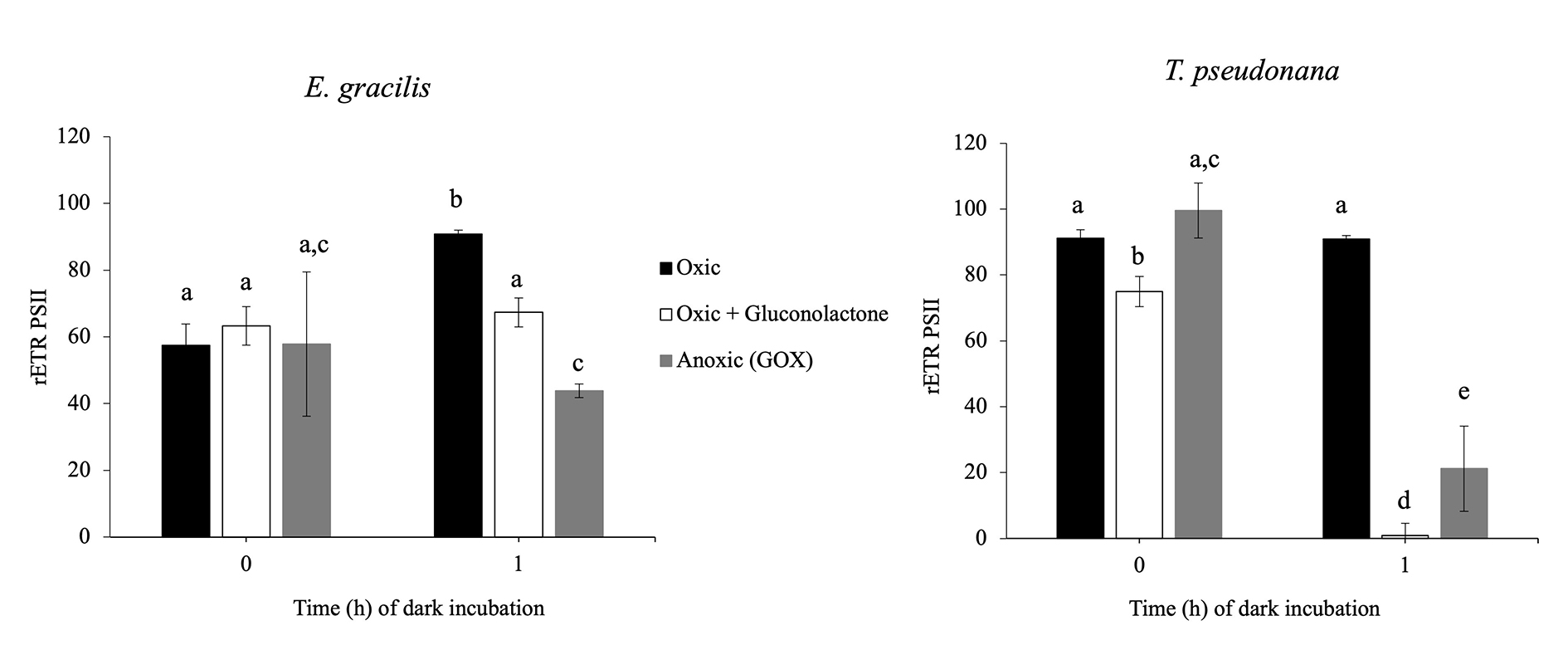

Supplement: Supplementary file 2 [file Image_1.jpg]

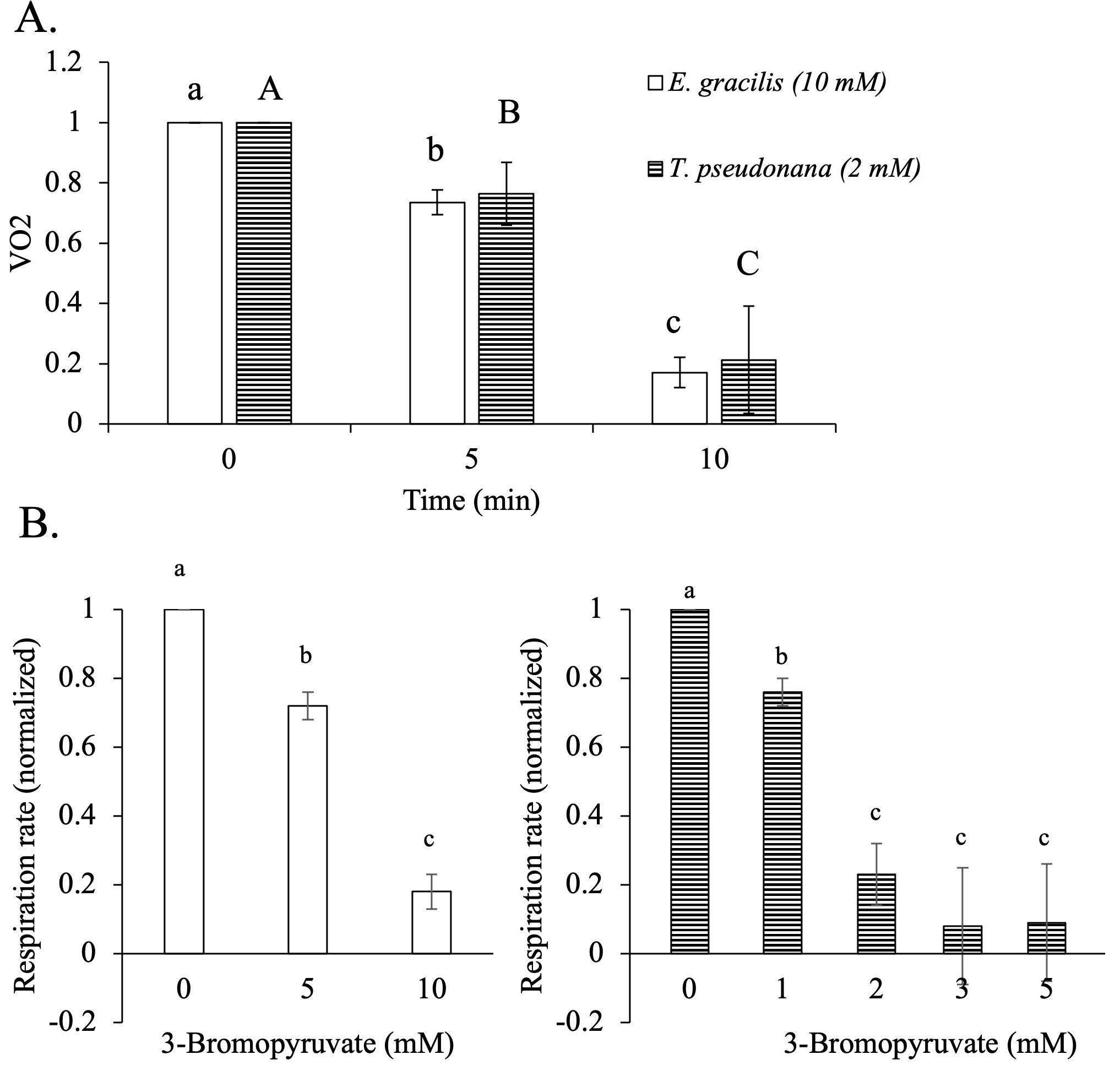

Supplement: Supplementary file 3 [file Image_2.jpg]

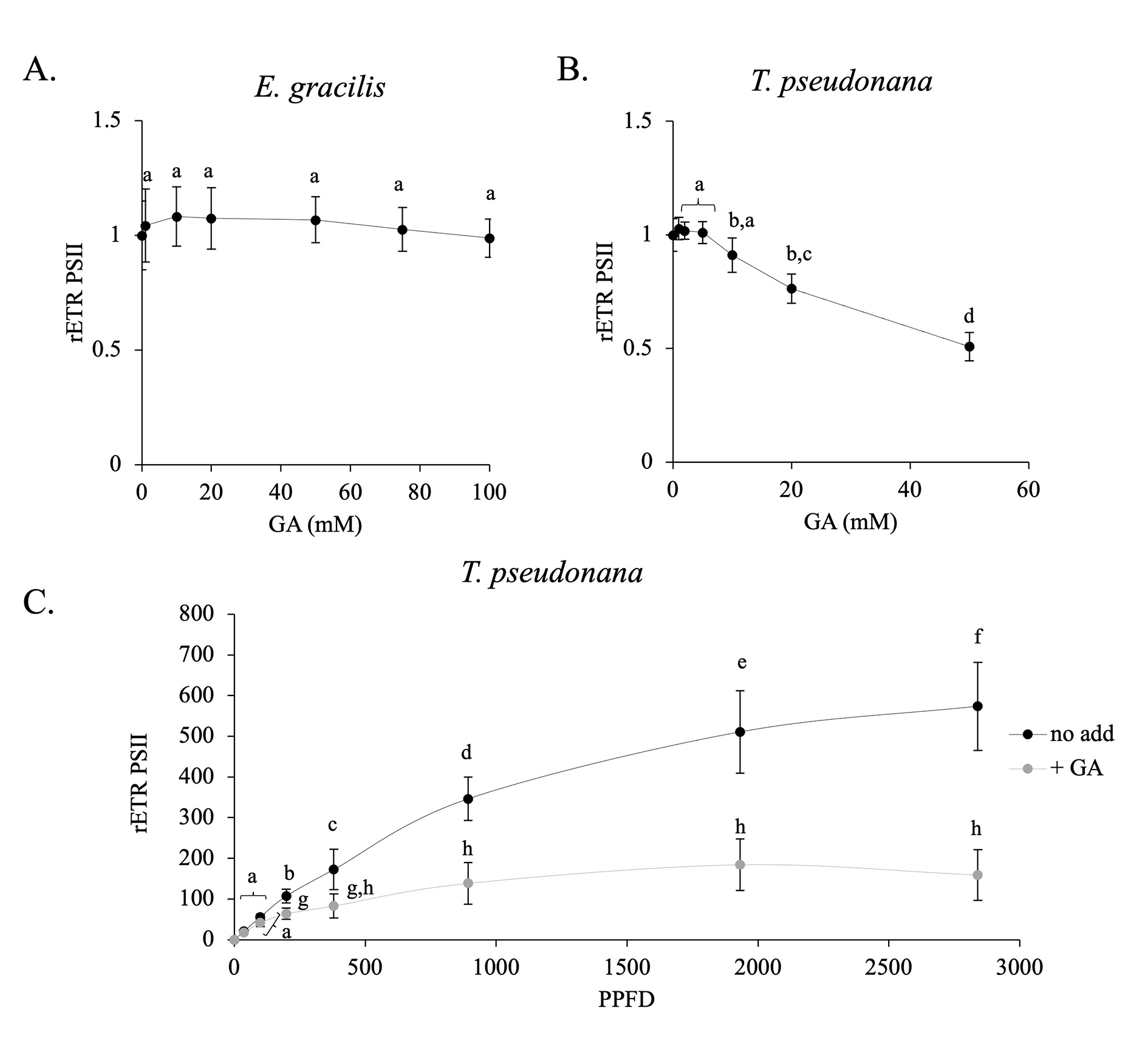

Supplement: Supplementary file 4 [file Image_3.jpg]
